# Repair of a Late Presentation Thoracic Aortic Aneurysm following Coarctation Repair

**DOI:** 10.1055/s-0042-1757950

**Published:** 2023-02-27

**Authors:** Robert J. Moon, Cristiano Spadaccio, Andrew J. Duncan, Mohamad N. Bittar

**Affiliations:** 1 Department of Cardiothoracic Surgery, Lancashire Cardiac Centre, Blackpool Victoria Hospital, Blackpool, United Kingdom

**Keywords:** aortic, aneurysm, coarctation, repair

## Abstract

We report the case of a 44-year-old gentleman who underwent coarctation repair at the age of 7 years. He was lost to follow-up and represented. Computed tomography scan demonstrated a 9.8-cm diameter aortic aneurysm involving the distal aortic arch and proximal descending aorta. Open surgery was performed to repair the aneurysm. The patient made an unremarkable recovery. He was followed up 12 weeks later, and significant improvement in preoperative symptoms was observed. This case demonstrates the importance of long-term follow-up.

## Introduction

We report the case of a 44-year-old gentleman who underwent coarctation of aorta (CoA) repair at the age of 7 years with the Dacron patch technique and was subsequently lost to follow-up.

## Case Presentation

The patient presented with hoarse voice, gastric reflux, and chronic cough over the prior 12 months. Historical notes were unavailable for review.

**Fig. 1:**
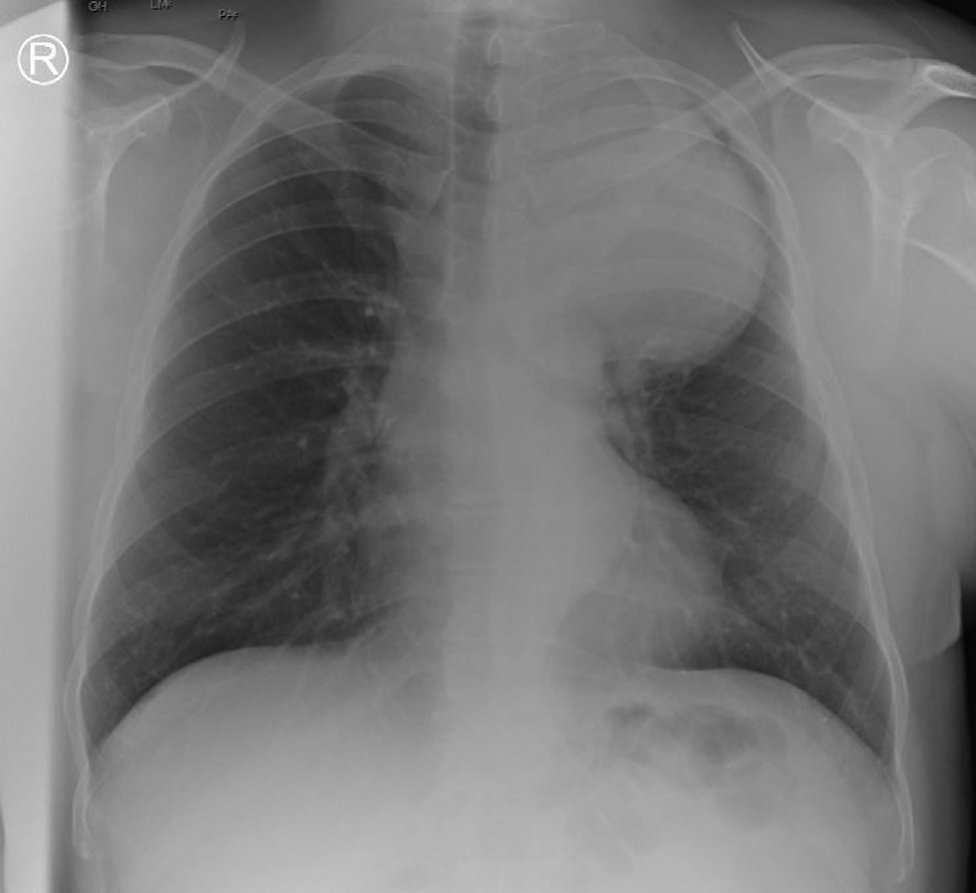
Chest X-ray showed evidence of a widened upper mediastinum with smooth contours.

**Fig. 2:**
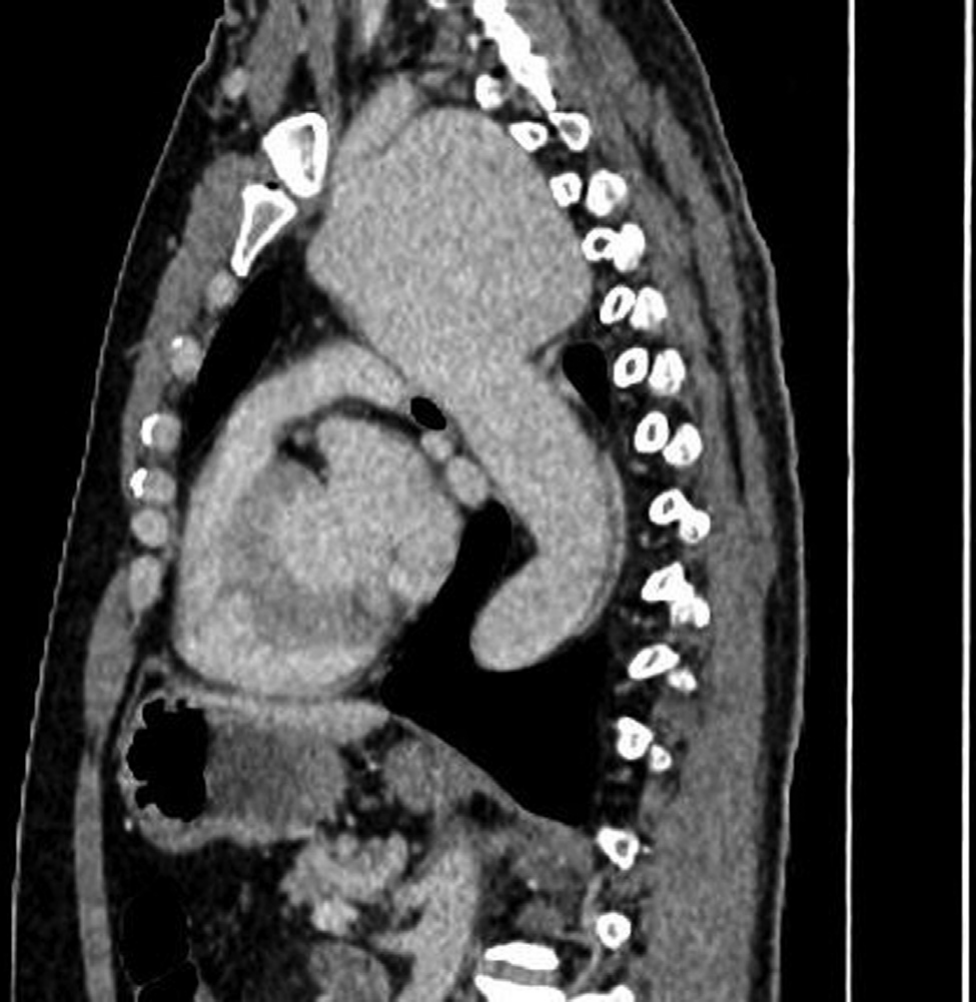
Left subclavian artery that arose from within the aneurysm.

A chest X-ray showed evidence of a widened upper mediastinum with smooth contours ( Fig. 1 ). This triggered a computed tomography (CT) aortogram which demonstrated a 9.8-cm diameter aortic aneurysm involving the distal aortic arch and proximal descending aorta. The aneurysm developed just distal to the origin of the left common carotid artery, and the left subclavian artery arose from within the aneurysm ( Fig. 2 ).

On CT imaging, the left vocal cord looked intact and was in paramedian position, therefore suggestive of left recurrent laryngeal nerve palsy resulting from extrinsic compression.

Echocardiogram confirmed a mildly dilated ascending aorta, tricuspid aortic valve, and mild aortic regurgitation with preserved heart function.

### Surgery and Follow-up

**Fig. 3:**
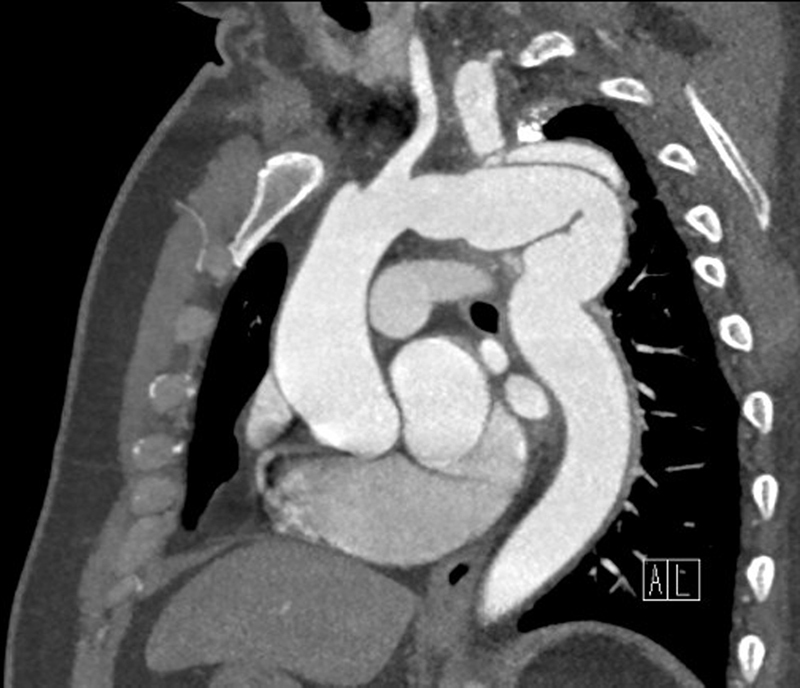
Repeat computed tomography scan confirming successful repair.

A left posterior–lateral thoracotomy was performed with deep hypothermic circulatory arrest. The aneurysmal mass, measuring over 10 cm in diameter, was dissected, excised, and replaced using size 28-mm Dacron tube graft with a single side arm which was anastomosed to the left subclavian artery. A split arterial line was used to continue upper and lower body circulation, while the distal aneurysm was being repaired. A direct left ventricular vent was used. Time of cardiopulmonary bypass was 165 minutes and deep hypothermic circulatory arrest (20°C) was 24 minutes. The patient made an unremarkable recovery and was discharged home 12 days later. When he was reviewed in the clinic 12 weeks postoperatively, both hoarseness and chronic cough had improved. Flexible nasendoscopy revealed a left vocal cord palsy which was most likely due to the original compression of the recurrent laryngeal nerves. Repeat CT scan confirmed successful repair. ( Fig. 3 ). The patient will be followed up with annual cardiac magnetic resonance imaging at our institution.

## Discussion

CoA is a congenital heart defect which accounts for 5 to 8% of all defects with 80% of these cases being male. Different surgical approaches to repair CoA can be utilized usually during childhood. These include end-to-end resection, patch repair, interposition grafts, and subclavian flap arterioplasty. Endovascular approaches are becoming an increasingly available option in appropriate cases. 1 Due to the size of the aneurysm, the experts felt that open repair in this case would give a better long-term solution. There was no safe landing zone for a stent.

Aneurysm formation is a recognized late complication of CoA repair and has been described after all types of intervention. Dacron patch repairs are associated with the highest incidence of postoperative aneurysm formation ranging between 33 and 51%. 2 The mechanism underlying this complication is not fully understood. However, factors related to patient age, residual unresected aortic pathological tissues, and operative techniques are claimed to be associated with this complication. True aneurysms tend to develop opposite to the site of the Dacron patch used to widen the aortic lumen and relieve stenosis. 3 However, pseudoaneurysms, dissecting aneurysms, or aortobronchial fistulae have also been described. 4 These conditions carry a significant mortality risk estimated to be between 36 and 100% if not diagnosed and treated. 5

The most reported presentation of post-CoA aneurysm repair is shortness of breath or back pain. Alternatively, it is detected during regular follow-up. Unlike previously reported literature, in this case, the main presentation was hoarseness as a result of external nerve compression. The size of 10 cm reached by the aneurysm has surely played a role in this patient's presentation. Importantly, to our knowledge, this is the largest post-CoA repair aneurysm described in the literature, with other authors reporting maximum diameters up to 60 mm. 6

Despite the progressively increasing use of endovascular approaches for postrepair complication of CoA, mainly for pseudoaneurysm, the vicinity to the subclavian artery and the size of the aneurysm made percutaneous stenting not a suitable option. As noted by others, while endovascular approaches could be a valid alternative in the cases of re-CoA, large-sized postrepair aneurysms are less amenable to be treated percutaneously. 6

Despite recommendations in the most recent guidelines, 7 the reality of long-term follow-up of patients operated on during childhood tends to be incomplete across the units and many patients are lost to follow-up. As a result, very late presentations of post-CoA repair aneurysms have been described.

The clinical relevance of this life-threatening condition should be carefully considered in the management of congenital heart disease patients, and this case strengthens the importance of a regular follow-up of these subjects. Ideally, patients should be registered with specialist services to ensure expert, life-long follow-up. Continuous lifelong surveillance is, therefore, mandatory, and cardiac magnetic resonance imaging 8 could represent a valuable tool in this young population, as avoiding radiation in patients of all ages.
